# Salivary cortisol as a non-invasive approach to assess stress in dystocic dairy calves

**DOI:** 10.1038/s41598-021-85666-9

**Published:** 2021-03-18

**Authors:** Levente Kovács, Fruzsina Luca Kézér, Szilárd Bodó, Ferenc Ruff, Rupert Palme, Ottó Szenci

**Affiliations:** 1 Institute of Animal Sciences, Hungarian University of Agriculture and Life Sciences, Guba Sándor utca 40, Kaposvár, 7400 Hungary; 2 Bovine Research Division, Bona Adventure Ltd, Peres utca 44, Gödöllő, 2100 Hungary; 3 Department of Methodology, Hungarian Central Statistical Office, Keleti Károly utca 5-7, Budapest, 1024 Hungary; 4 Unit of Physiology, Pathophysiology, and Experimental Endocrinology, University of Veterinary Medicine, Veterinärplatz 1, 1210 Vienna, Austria; 5 Department of Obstetrics and Food Animal Medicine Clinic, University of Veterinary Medicine, István utca 2, Budapest, 1078 Hungary

**Keywords:** Physiology, Zoology, Environmental sciences

## Abstract

The intensity and the magnitude of saliva cortisol responses were investigated during the first 48 h following birth in newborn dairy calves which underwent normal (eutocic, **EUT**, n = 88) and difficult (dystocic, **DYS**, n = 70) calvings. The effects of parity and body condition of the dam, the duration of parturition, the time spent licking the calf, the sex and birth weight of the calf were also analyzed. Neonatal salivary cortisol concentrations were influenced neither by factors related to the dam (parity, body condition) nor the calf (sex, birth weight). The duration of parturition and the time spent licking the calf also had no effect on salivary cortisol levels. Salivary cortisol concentrations increased rapidly after delivery in both groups to reach their peak levels at 45 and 60 min after delivery in EUT and DYS calves, respectively supporting that the birth process means considerable stress for calves and the immediate postnatal period also appears to be stressful for newborn calves. DYS calves exhibited higher salivary cortisol concentrations compared to EUT ones for 0 (*P* = 0.022), 15 (*P* = 0.016), 30 (*P* = 0.007), 45 (*P* = 0.003), 60 (*P* = 0.001) and 120 min (*P* = 0.001), and for 24 h (*P* = 0.040), respectively. Peak levels of salivary cortisol and the cortisol release into saliva calculated as AUC were higher in DYS than in EUT calves for the 48-h of the sampling period (*P* = 0.009 and *P* = 0.003, respectively). The greater magnitude of saliva cortisol levels in DYS calves compared to EUT ones suggest that difficult parturition means severe stress for bovine neonates and salivary cortisol could be an opportunity for non-invasive assessment of stress during the early neonatal period in cattle.

## Introduction

Bovine parturition is initiated by rising cortisol levels in the fetus that provoke a cascade of endocrine activity in the dam^1^. This increase in fetal cortisol is a result of increased adrenocorticotropic hormone production by the maturing fetal pituitary caused by fetal stressors such as hypoxia and hypercapnia. However, the process of parturition may also be a stressful event for the fetus, especially during the stage of expulsion, if difficulties at calving occur^2^.

Acute responses to stressful stimuli include activation of the hypothalamic–pituitary–adrenal (**HPA**) axis and the autonomic nervous system. Pain biomarkers related to HPA axis are often measured in biological samples (e.g. blood). Plasma cortisol concentrations have been widely used to evaluate the HPA axis activity in painful procedures in calves^3^ and in mature cattle^4,5^. However, the process of taking blood samples is accompanied by additional stress, which can affect the test results^6^. According to human studies, psychobiological mechanisms, which trigger the HPA axis, can be assessed by salivary cortisol concentrations^7,8^ that reflect unbound (free) cortisol^9,10^. Saliva samples can be easily taken at fixed time intervals after an imposed stress^11^ and it is a minimally invasive^6^ and appropriate method to assess HPA axis reactivity in cattle. Furthermore, salivary cortisol correlates well with plasma cortisol with 0^12^ or with a 10 min time lag^13,14^.

Although dystocia is a growing problem on dairy farms^15^, it is not known how it influences the stress level of calves during the first 48 h of life which is the most critical period in terms of survival^16^. The present paper attempts to look at the effects of dystocia and some calving-related factors (i.e. parity and body condition of the dam, the duration of parturition, the time spent with licking the calf, and the sex and birth weight of the calf) on salivary cortisol as noninvasive measure of HPA activity in newborn calves.

## Methods

### Experimental farm and animals

All methods and the applied procedures on the animals were performed in accordance with the relevant guidelines and regulations of the Pest County Government Office, Department of Animal Health (Permit Number: PE/EA/1973-6/2016) that approved the study. A total of 168 calvings were enrolled on a large-scale dairy farm in Hungary consisting of 1,200 lactating Holstein–Friesian cows. The farm was visited for an 8-month period in the spring (between February and May) and autumn (September and December) of 2016 to exclude the possible effects of heat stress on the trial results. Cows calved in the prepartum group pen or, if assistance was required, in a separate maternity pen. Calves were removed from the dams 2 h after birth and received colostrum by nipple bottle and then fed four times a day with 1.65 L of fresh-cow colostrum per feeding during the first 48 h of life. Newborns were housed individually until 60 d of age in 1.65 × 1.20 m plastic calf hutches with a 1.60 m^2^ exercise pen, both bedded with straw.

### Observation of calvings

Calvings occurring in the group pen were observed with two day/night outdoor network bullet cameras (Vivotek IP8331, VIVOTEK Inc., Taipei, Taiwan), while individual calvings were observed with two portable video cameras (Legria HF M36, CANON Inc., Japan, Tokyo).

Dystocia (**DYS**, n = 70) was defined as calving difficulty resulting from prolonged spontaneous calving (> 2 h from the appearance of hooves to delivery) or prolonged or severe assisted extraction by one or more people with considerable force with a calving rope or with a calving jack^17^. Normal calving (eutocia; **EUT**, n = 98) was regarded as a combination of ‘no assistance’ and ‘slight assistance’ (where assistance was brief, and traction was slight) by one person^17^.

The condition of the dam was scored using the 5-point BCS system^18^ following calving. Sex and birth weight of the calves were also recorded immediately after delivery. Since during stage 1 of labor significant stress was found in dairy cows^19^, and thus, possibly for newborn calves, the duration of parturition was considered as the time lag between the onset of stage 1 (the onset of calving restlessness) and the completion of stage 2 of labor (delivery). The onset of calving restlessness was determined based on accepted behavioral predictors such as lying down frequency, tail raising and walking^20^ and was observed by two trained experimenters through the above-mentioned camera system. The time spent licking the calf’s head or body was recorded during the first 2 h following calving according to the recommendation of Jensen^21^.

### Salivary cortisol

Using a synthetic swab (Salivette Cortisol, Sarstedt, Nümbrecht-Rommelsdorf, Germany), saliva samples were taken 0, 15, 30, 45, 60, 120 min, 24 and 48 h after delivery. Without retain of the animals, the swabs were placed loosely onto the tongue of the calf until it was well soaked with saliva. This procedure required up to 10 s, and the animals tolerated saliva samplings well. The swabs were then inserted into Salivette polypropylene tubes, which were placed on ice immediately after sampling and stored at 4 °C until centrifugation (within 10 min after sampling) at 1000 g for 10 min. At least 1.5 ml saliva per sample was obtained and frozen at − 20 °C until analysis. After a further dilution step (1:10) with assay buffer, salivary cortisol concentrations were determined in an aliquot (10 µl) with a competitive cortisol enzyme immunoassay (EIA). For the details of the EIA, including cross-reactions and its application in calves refer to Palme and Möstl^22^ and Wagner et al.^23^. Interassay coefficients of variation of high and low concentration pool samples from saliva of the calves of this study were 9.2% and 12.8%, respectively. The detection limit of the assay was 0.02 ng/ml.

### Statistical analysis

Statistical analyses were performed in the R–3.3.1 statistical environment and language^24^. All results are expressed as mean plus SEM values.

Multivariable linear regression models were fit to the data^25^ for each sampling time point to test the effects of independent variables on salivary cortisol concentrations. Independent variables were parity and BCS of the dam, sex and birth weight of the calf, the duration of parturition, calving ease (dystocia or eutocia), and the time spent licking the calf. Salivary cortisol concentrations were inserted into the models as response (dependent) variables. Log-transformation of saliva cortisol concentrations was applied to satisfy the normality and variance homogeneity assumptions of the models.

Based on the results of the linear models (only calving ease had a significant effect on cortisol levels; see Results section), for reducing the number of statistical comparisons between groups during the 48-h postnatal period, salivary cortisol concentrations of EUT and DYS calves were calculated as area under the curve (**AUC**) and cortisol responses were compared. The AUC represents both the magnitude and the changes over time of the response^26^. Response parameters included peak values of salivary cortisol concentrations, baseline (48 h sample), and AUCs that were determined for the first 48 h of life following delivery utilizing a trapezoid method described by Lay et al.^27^ as follows:

$$ {\text{AUC}}_{{{\text{RESP}}}} = \, \Sigma \left[ {\left( {{\text{P}}_{{\text{n}}} + {\text{P}}_{{{\text{n}} + {1}}} } \right)/{2 } \times {\text{ m }}{-}{\text{ BASELINE}}} \right], $$

where ‘P’ is salivary cortisol concentration at a given time point, ‘m’ is the time in minutes between the two P values and ‘baseline’ is the mean value of cortisol concentrations in saliva 48 h after delivery. Data were tested for constant variance (Levene’s test) and the Shapiro–Wilk test was used for testing the equality of error variances. Comparisons between EUT and DYS groups for peak salivary cortisol levels and AUCs were made by a Wilcoxon rank-sum test. Significance was set at the level of 0.05 in case of both parameters.

Non-significant variables on salivary cortisol concentrations were compared between EUT and DYS groups with the Welch’s two-sample *t* test (parity and BCS of the dam, sex and birth weight of the calf, the duration of parturition, and the time spent licking the calf) and with the Pearson’s Chi-squared test (proportions of male and female calves) at the significance level of 0.05 in both cases.

## Results

From the 168 calvings, 49, 56 and 63 calves were born to first, second and third parity cows, respectively. Comparison of independent variables between EUT and DYS groups is shown in Table 1. Salivary cortisol concentrations determined within 48 h after delivery were neither influenced by factors related to the dam (parity, body condition score, **BCS**) nor the calf (sex, birth weight). Although the duration of parturition (range: 1.3–8.2 h) and the time spent linking the calf (5.5–86.5 min) differed significantly between EUT and DYS calves (Table 1) none of these factors influenced salivary cortisol levels.

**Table 1:** Characteristics of calvings involved in this study (means ± SEM).

Calving category	BCS of the dam^1^	Parity of the dam	Birth weight of the calf (kg)	Sex of the calf		Duration of calving (min)^2^	Time spent licking the calf (min/2 h)
				Male	Female		
Eutocic (n = 98)	3.2 ± 0.1	2.3 ± 0.2	37.8 ± 1.0	n = 38 (38.8%)	n = 60 (61.2%)	138.4 ± 34.5	68.3 ± 35.4
Dystocic (n = 70)	3.3 ± 0.1	2.1 ± 0.2	40.2 ± 1.2	n = 27 (38.6%)	n = 43 (61.4%)	242.7 ± 66.8	46.2 ± 23.6
*P* value	0.850	0.345	0.560	1.00	1.00	0.020	0.035

^1^BCS of the dam was scored using the 5-point USA scoring system^18^ following calving.

^2^Between the onset of calving restlessness and delivery (including stages 1 and 2 of labor).

Statistical significances are based on the Welch’s two-sample t test in cases of BCS and parity of the dam, birth weight of the calf, duration of calving and time spent licking the calf. The proportions of male and female calves were compared between groups with the Pearson’s Chi-squared test.

**Figure 1:**
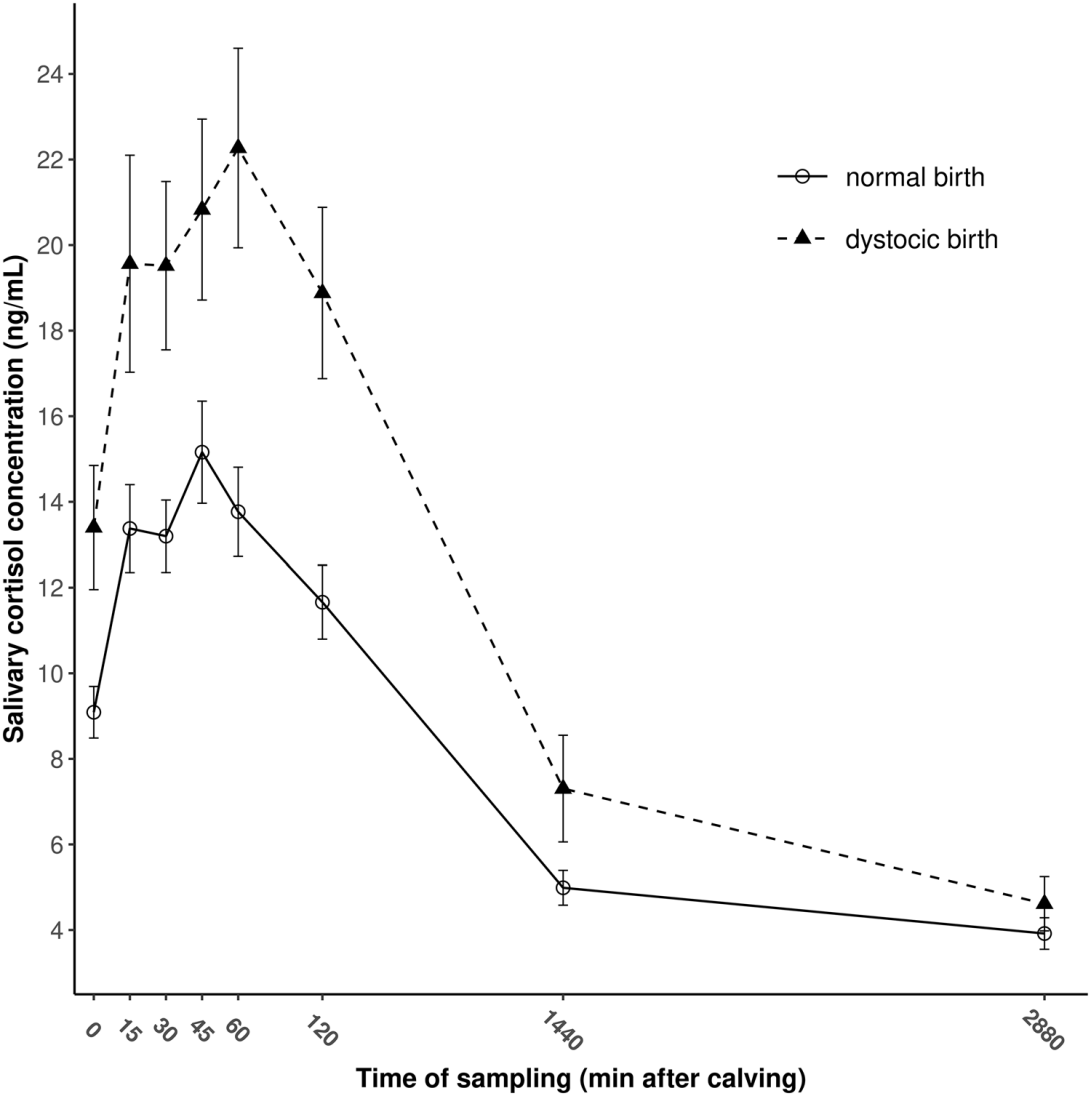
The evolution of salivary cortisol concentrations during the first 48 h of life in dairy calves born to eutocic (n = 98) and dystocic (n = 70) dams. Data are given in means ± SEM.

Except for 48 h after delivery, linear models (df = 7; 150) indicated higher salivary cortisol concentrations in DYS calves compared to EUT ones for 0 (*P* = 0.022), 15 (*P* = 0.016), 30 (*P* = 0.007), 45 (*P* = 0.003), 60 (*P* = 0.001) and 120 min (*P* = 0.001), and for 24 h (*P* = 0.040) after birth, respectively. The evolution of salivary cortisol concentrations after delivery are shown in Fig. 1 for EUT and DYS calves. The HPA response showed a similar pattern in both groups. Salivary cortisol concentrations increased rapidly after delivery in both groups to reach their peak levels at 45 and 60 min after delivery in EUT and DYS calves, respectively. Afterward, a gradual decrease in cortisol concentrations was observed in both groups (Fig. 1). Twenty-four h post-calving, salivary cortisol decreased to 32.9% and 33.7% of the peak levels in EUT and DYS calves, respectively and for the 48-h samples, similar circulating cortisol concentrations were observed in both groups in saliva (*P* = 0.245).

DYS calves exhibited significantly higher peak levels and AUC of salivary cortisol than EUT calves (with 45.6% and 92.1%, respectively) for the 48-h of the sampling period (Table 2).

**Table 2:** Peak levels of salivary cortisol concentrations and salivary cortisol concentrations calculated as area under the curve (AUC) of newborn calves born from eutocic (EUT, n = 98) and dystocic (DYS, n = 70) deliveries.

Salivary cortisol	Unit	Group		Statistics^b^	
		EUT	DYS	W	*P* value
Peak levels	ng/mL	21.7 ± 1.3 (6.7–42.6)	31.6 ± 2.9 (12.4–78.8)	2159	0.009
AUC^a^	ng/mL × min	7988 ± 1078 (560.7–37 354)	15 346 ± 2554 (2561–106 428)	2075	0.003

Descriptive statistics are based on means ± SEM (ranges) of non-transformed data.

^a^Area under the curve was calculated for the first 48 h after delivery.

^b^Statistical significances for response parameters of the HPA axis are based on results from the Wilcoxon rank-sum test with continuity correction. W = Wilcoxon value; the sum of the ranks in one of both groups.

## Discussion

This is the first study which investigates both the intensity and magnitude of the postnatal HPA response to birth of EUT and DYS dairy calves using AUC analyses based on high sampling frequencies of saliva. The present findings demonstrate that the birth process induces significant elevation in HPA axis activity in newborn calves, even if no difficulties during parturition occur. Differences between cortisol levels measured at 0 and 48 min in EUT (106.4%) and DYS calves (175.2%) found in the present study suggest that calves experienced stress before delivery irrespective of obstetrical conditions. However, calves experiencing DYS births exhibited greater saliva cortisol levels, thus higher stress after calving compared to EUT calves. Our results support earlier findings on serum^28^, plasma^29^ and salivary cortisol levels^30^ of EUT and DYS calves. In general, peak cortisol levels found in saliva in the present study were similar to those observed by Stewart et al.^31^ in plasma 40 min after administration of adrenocorticotropic hormone (34.5 ng/mL), or after castration without local anesthetic (28.7 ng/mL) in Holstein–Friesian heifer calves^32^.

An earlier study found lower peak levels of salivary cortisol (14.8 ng/mL) in newborn calves after induced parturitions^33^, whereas others reported 6 ng/mL concentrations in calves born from assisted deliveries^30^; however, authors collected saliva once within 24 h of birth, therefore they were not able to determine peak levels.

Gradually increasing cortisol levels after delivery in both groups support that the birth process means considerable stress for calves^34^ and the immediate postnatal period also appears to be stressful for the newborn calf. In the present study, peak cortisol levels at 45 and 60 min after delivery in EUT and DYS calves, respectively, may be a delayed increase due to cortisol transfer from the serum to the saliva^13,14^ or even reflect additional stress experienced by the calves during transition from the fetal to the extrauterine life.

Similar to our findings, Nagel et al.^33^ reported peak saliva cortisol levels at 60 min after birth. Others found peak levels immediately after birth in serum^28^, and 3 h after delivery in plasma^35^. Salivary cortisol peak levels found in the present study in DYS calves was 12.2% of those observed by the latter authors in plasma^35^, which support field^12^ and laboratory observations on cattle^36^ indicating that salivary cortisol levels yield around 10% of plasma cortisol levels.

Although Hoyer et al.^37^ found that reversal of stress occurs rapidly during the first hours of neonatal life, the results presented here suggest that newborn calves appear to adapt to the extrauterine environment by 24 h of age. In line with our results, cortisol concentrations measured from saliva^33^ and plasma^35,38,39^ decreased gradually for 24 and 48 h after parturition.

As calving means significant stress also for the dam even from the onset of stage 1 of labor^19^, it came into question if naturally occurring cortisol in cows before delivery had a significant influence on the amount of cortisol levels of the neonatal calf. It has been shown in goats^40^ and ewes^41^ that cortisol can partly cross the placenta, from the mother to the fetus and may lead to hypercortisolism in situations of prolonged stress experienced by the dam during parturition^42^. However, results of Wooley^35^ indicate that maternal cortisol concentrations in plasma do not influence calf cortisol concentration in cattle.

It should be noted that within the early neonatal life, other factors might also affect HPA axis functioning. Similar to recent observations^33^, birth weight and duration of parturition had no effect on neonatal salivary cortisol concentrations. As calves were removed from the dams only 2 h after delivery to receive colostrum by farmhand, the only factor would have been the dam-offspring contact. According to our earlier findings, the duration of licking the calf is a prominent factor in the thermal and metabolic adaptation of newborn calves to extrauterine life^43^. Although the time spent licking the calf had no effect on salivary cortisol concentrations in this study, it can be assumed that maternal grooming might have caused a positive stress for the calves by increasing cortisol levels between 15 and 60 min after birth, irrespectively for calving ease.

As a progressive maturation and activation of the fetal HPA axis during late gestation results in a considerably increased cortisol release from the fetal adrenals starting between 7 and 3 days before parturition^44,45^ it is thus questionable whether this initial fetal cortisol would affect cortisol levels measured from saliva after delivery. According to our assumption it could not have been significantly present in the saliva of newborns, as fetal cortisol is proven to be absorbed by the maternal unit causing initiation of the preparation stage of labor^46^ and the gradual prepartum rise in fetal plasma cortisol during the last week of gestation was found to be much less marked even in spontaneously born calves than the abrupt increase immediately after birth^47^.

Glucocorticoids can have a significant influence on the amount of immunoglobulins in colostrum and also on the amount of immunoglobulins absorbed by the neonate. Decreased cortisol concentrations may reduce the ability or time available for the calf to absorb colostral immunoglobulins, whereas increased serum cortisol concentrations increase IgG concentrations^48^. However, it has been proposed that the increased susceptibility to bacterial infection in calves may be enhanced by high plasma cortisol concentrations at birth and their effects upon the lymphocytes^49^. This is a limitation of the present study that we did not measure immune parameters or followed-up calves to examine the longer-term effects of dystocia-related stress either on growth, behavior, or overall welfare.

The greater magnitude of saliva cortisol responses in DYS calves compared to EUT ones suggest that difficult calving is more stressful for bovine neonates than a normal birth due to prolonged parturition and/or forced extraction, and salivary cortisol could be an opportunity for non-invasive assessment of stress during the early neonatal period in cattle. The findings of the present study should be integrated in further investigations with data from behavioral observations, production, and pathology records in a comprehensive approach of bovine neonatal well-being.

## Data Availability

All materials, data that support the findings of this study and associated protocols are available from the corresponding author upon reasonable request.

## References

[1] Hunter JT, Fairclough RJ, Peterson AJ, Welch RA (1977). Foetal and maternal hormonal changes preceding normal bovine parturition. Acta Endocrinol..

[2] Kovács L, Kézér FL, Szenci O (2016). Effect of calving process on the outcomes of delivery and postpartum health of dairy cows with unassisted and assisted calvings. J. Dairy Sci..

[3] Stafford KJ, Mellor DJ (2011). Addressing the pain associated with disbudding and dehorning in cattle. Appl. Anim. Behav. Sci..

[4] Lay DC (1992). Behavioral and physiological effects of freeze or hot-iron branding on crossbred cattle. J. Anim. Sci..

[5] Fidan A, Pamuk FK, Ozdemir A, Saritas ZK, Tarakci U (2010). Effects of dehorning by amputation on oxidant-antioxidant status in mature cattle. Rev. Med. Vet..

[6] Cook NJ (2012). Review: Minimally invasive sampling media and the measurement of corticosteroids as biomarkers of stress in animals. Can. J. Anim. Sci..

[7] Hellhammer DH, Wüst S, Kudielka BM (2009). Salivary cortisol as a biomarker in stress research. Psychoneuroendocrinology.

[8] Kudielka BM, Hellhammer DH, Wüst S (2009). Why do we respond so differently? Reviewing determinants of human salivary cortisol responses to challenge. Psychoneuroendocrinology.

[9] Kirschbaum C, Fink G (2000). Salivary cortisol. Encyclopedia of Stress.

[10] Möstl E, Palme R (2002). Hormones as indicators of stress. Domest. Anim. Endocrinol..

[11] Mormède P (2007). Exploration of the hypothalamic-pituitary-adrenal function as a tool to evaluate animal welfare. Physiol. Behav..

[12] Kovács L (2016). Hypothalamic–pituitary–adrenal and cardiac autonomic responses to transrectal examination differ with behavioral reactivity in dairy cows. J. Dairy Sci..

[13] Negrao JA, Porcionato MA, de Passille AM, Rushen J (2004). Cortisol in saliva and plasma of cattle after ACTH administration and milking. J. Dairy Sci..

[14] Hernandez CE (2014). Time lag between peak concentrations of plasma and salivary cortisol following a stressful procedure in dairy cattle. Acta Vet. Scand..

[15] Mee JF (2008). Newborn dairy calf management. Vet. Clin. N. Am. Food Anim. Pract..

[16] Schuijt G (1990). Iatrogenic fractures of ribs and vertebrae during delivery in perinataly dying calves: 235 cases (1978–1988). J. Am. Vet. Med. Assoc..

[17] Mee JF, Berry DP, Cromie AR (2011). Risk factors for calving assistance and dystocia in pasture-based Holstein-Friesian heifers and cows in Ireland. Vet. J..

[18] Hady PJ, Domecq JJ, Kaneene JB (1994). Frequency and precision of body condition scoring in dairy cattle. J. Dairy Sci..

[19] Kovács L (2015). Heart rate and heart rate variability in multiparous dairy cows with unassisted calvings in the periparturient period. Physiol. Behav..

[20] Miedema HM, Cockram MS, Dwyer CM, Macrae AI (2012). Behavioural predictors of the start of normal and dystocic calving in dairy cows and heifers. Appl. Anim. Behav. Sci..

[21] Jensen MB (2012). Behaviour around the time of calving in dairy cows. Appl. Anim. Behav. Sci..

[22] Palme R, Möstl E (1997). Measurement of cortisol metabolites in faeces of sheep as a parameter of cortisol concentration in blood. Z. Saugetierkd. Int. J. Mammal. Biol..

[23] Wagner K (2013). Mother rearing of dairy calves: Reactions to isolation and to confrontation with an unfamiliar conspecific in a new environment. Appl. Anim. Behav. Sci..

[24] R Core Team. *R: A Language and Environment for Statistical Computing*. R Foundation for Statistical Computing, Vienna. http://www.r-project.org/ (2017).

[25] Pinheiro JC, Bates DM (2000). Mixed-Effects Models in S and S-PLUS.

[26] Fekedulegn DB (2017). Area under the curve and other summary indicators of repeated waking cortisol measurements. Psychosom. Med..

[27] Lay DC (1996). Adrenocorticotropic hormone dose response and some physiological effects of transportation on pregnant Brahman cattle. J. Anim. Sci..

[28] Vannucchi CI (2015). Association between birth conditions and glucose and cortisol profiles of periparturient dairy cows and neonatal calves. Vet. Rec..

[29] Civelek T, Celik HA, Avci G, Cingi CC (2008). Effects of dystocia on plasma cortisol and cholesterol levels in holstein heifers and their newborn calves. Bull. Vet. Inst. Pulawy.

[30] Barrier AC (2013). The impact of dystocia on dairy calf health, welfare, performance and survival. Vet. J..

[31] Stewart M, Stafford KJ, Dowling SK, Schaefer AL, Webster JR (2008). Eye temperature and heart rate variability of calves disbudded with or without local anaesthetic. Physiol. Behav..

[32] Stewart M, Verkerk GA, Stafford KJ, Schaefer AL, Webster JR (2010). Noninvasive assessment of autonomic activity for evaluation of pain in calves, using surgical castration as a model. J. Dairy Sci..

[33] Nagel C (2016). Stress response and cardiac activity of term and preterm calves in the perinatal period. Theriogenology.

[34] Aurich JE (1993). Influence of labor and neonatal hypoxia on sympathoadrenal activation and methionine enkephalin release in calves. Am. J. Vet. Res..

[35] Wooley, D. N. Prepartum maternal cortisol concentrations on postnatal cortisol concentration and immunoglobulin absorption in neonatal dairy calves. LSU Master's Theses. 4124. https://digitalcommons.lsu.edu/gradschool_theses/4124 (2010).

[36] Chacón G, Laita SG-B, del Portal JCI, Liesa JP (2004). Validation of an EIA technique for the determination of salivary cortisol in cattle. Spanish J. Agric. Res..

[37] Hoyer C, Grunert E, Jochle W (1990). Plasma glucocorticoid concentrations in calves as an indicator of stress during parturition. Am. J. Vet. Res..

[38] Hadorn U, Hammon H, Bruckmaier RM, Blum JW (1997). Delaying colostrum intake by one day has important effects on metabolic traits and on gastrointestinal and metabolic hormones in neonatal calves. J. Nutr..

[39] Hammon HM, Blum JW (1998). Metabolic and endocrine traits of neonatal calves are influenced by feeding colostrum for different durations or only milk replacer. J. Nutr..

[40] Thorburn GD, Nicol DH, Bassett JM, Shutt DA, Cox RI (1972). Parturition in the goat and sheep: changes in corticosteroids, progesterone, oestrogens and prostaglandin F. J. Reprod. Fert..

[41] Dixon R (1970). Feto-maternal transfer and production of cortisol in the sheep. Steroids.

[42] Jones CT, Robinson RO, Luther E, Ritchie JWK, Worthington D (1977). Control of adrenocorticotrophin secretion by catecholamines in the pregnant and foetal sheep. J. Endocr..

[43] Kovács L, Kézér FL, Albert E, Ruff F, Szenci O (2017). Seasonal and maternal effects on acid-base, lactate, electrolyte and hematological status of 205 dairy calves born to eutocic dams. J. Dairy Sci..

[44] Schuler G, Fürbass R, Klisch K (2018). Placental hormones in gestation and parturition. Anim. Reprod..

[45] Taverne MAM, Bevers MM, van der Weyden GC, Dieleman SJ, Fontijne P (1988). Concentration of growth hormone, prolactin, and cortisol in fetal and maternal blood and amniotic fluid during late pregnancy and parturition in cows with cannulated fetuses. Anim. Reprod. Sci..

[46] Hoffmann B, Wagner WC, Rattenberger E, Schmidt J (1977). Endocrine relationships during late gestation and parturition in the cow. Ciba Found. Symp..

[47] Comline RS, Hall LW, Lavelle RB, Nathanielsz PW, Silver M (1974). Parturition in the cow: endocrine changes in animals with chronically implanted catheters in the foetal and maternal circulations. J. Endocrinol..

[48] Johnston NE, Oxender WD (1979). Effect of altered serum glucocorticoid concentrations on the ability of the newborn calf to absorb colostral immunoglobulin. Am. J. Vet. Res..

[49] Eberhart RJ, Patt JA (1971). Plasma cortisol concentrations in newborn calves. Am. J. Vet. Res..

